# The Causes of Typhoid Fever

**Published:** 1875-12

**Authors:** 


The Causes of Typhoid Fever.—Dr. Edwin M. Snow, in his last report as City Registrar of Providence, says of this disease:

"In regard to the causes of the fever, in one instance well-water seemed to be the undoubted cause of several cases; but it is certain that, of the whole number of descendents from fever, two-thirds were of American parentage. Those who are familiar with the localities in which the fever prevails, and with the far worse localities that are comparatively exempt from it, must be satisfied that sink-drains and cesspools are not the chief causes, nor even the prominent of this particular disease in this city.—*Med. and Surg. Reporter.*

